# Anaesthetic Considerations in Paediatric Myasthenia Gravis

**DOI:** 10.4061/2011/250561

**Published:** 2011-09-25

**Authors:** Oliver William Masters, Oliver N. Bagshaw

**Affiliations:** Birmingham Children's Hospital, Birmingham B4 6NH, UK

## Abstract

Myasthenia gravis is of particular interest to anaesthetists because of the muscle groups affected, the pharmacology of the neuromuscular junction, and interaction of both the disease and treatment with many anaesthetic drugs. Anaesthetists may encounter children with myasthenia either to facilitate treatment options or to institute mechanical ventilation in the face of a crisis. This paper reviews the literature pertaining to the pathophysiology and applied pharmacology of the disease and explores the relationship between these and the anaesthetic management. In addition to illustrating the tried-and-tested techniques, some newer management options are explored.

## 1. Introduction

Myasthenia gravis (MG) is an autoimmune disease of the neuromuscular junction characterised by weakness and fatiguability of skeletal muscles. It can present significant challenges to the anaesthetist, not only because of the nature of the disease itself but also because of the treatment that patients may be on and the interaction of these treatments with many anaesthetic drugs. 

MG affects children in three main forms—juvenile, neonatal, and congenital. Juvenile is the commonest form of the disease representing 10–15% of all cases of myasthenia in both children and younger adults [1]. It is caused by IgG autoantibodies which are directed towards the acetylcholine receptors on the postsynaptic membrane of the neuromuscular junction. This causes both a decrease in receptor numbers as well as a conformational change in the motor endplate, resulting in fewer synaptic folds and a wider synaptic cleft [2]. As a consequence, the usual physiological excess of acetyl choline is deranged and a nerve action potential may not result in muscular contraction. In juvenile MG the thymus gland is often hyperplastic, and although the exact relationship between the gland and the disease remains obscure, certain patients are known to benefit from thymectomy [3, 4].

Neonatal MG affects 10% of babies born to mothers suffering from MG and is caused by the placental transfer of maternal antiacetylcholine receptor antibodies. Recovery tends to occur spontaneously within about 6 weeks. In some cases mechanical ventilation and treatment with anticholinesterases may be required [5].

Congenital MG is a rare heterogeneous disease which can affect the whole neuromuscular junction. It is classified into presynaptic, synaptic, and postsynaptic forms, and treatment and clinical picture depend on the subtype [6]. Owing to its rarity and the fact that anaesthetic experience is extremely limited, we shall not consider it further. In this paper we shall concentrate on the juvenile form of the disease as this is the form which is most likely to be encountered by anaesthetists.

Anaesthetic management depends upon sound understanding of the pathophysiology of the disease process as well as the pharmacology of the neuromuscular junction.

## 2. Clinical Picture

The clinical picture is one of weakness and fatiguability of skeletal muscles. A common presentation is of ocular weakness resulting in ptosis, diplopia, or variable strabismus. Presentation is however variable and may involve ocular signs, generalised weakness, or a combination [7–10]. Bulbar weakness is often a prominent feature and may result in feeding difficulties as well as dysarthria. Respiratory muscles are affected in about 20% of myasthenic patients though rarely in isolation [5]. Myasthenic crises (see below) may occur during times of physiological stress such as infection, surgery, or heightened emotion and result in an acute deterioration in muscle strength necessitating mechanical ventilation [11].

## 3. Diagnosis

Confirmation of a diagnosis involves 3 tests [11–13]. Administration of edrophonium—a short acting acetylcholinesterase inhibitor—inhibits the breakdown of acetylcholine at the neuromuscular junction (NMJ). Thus, the physiological excess of acetylcholine is restored. The test, if positive, results in a rapid improvement in muscle strength which occurs after about 30 s and lasts for about 5 minutes. This is often known as the Tensilon test. Antibodies to the acetylcholine receptor itself can be detected in the blood of certain individuals with MG. The test is highly specific and becomes increasingly sensitive with increasing age and increasing duration of disease such that about 56% of prepubertal children and up to 82% of peripubertal children with juvenile MG will have detectable antibodies against the acetylcholine receptor [14]. Single fibre EMG is a definitive way of elucidating neuromuscular transmission disorders but is not specific for MG and may well not be tolerated in children [15].

## 4. Treatment

There are 2 main approaches to the treatment of children with myasthenia gravis [16]. The first strategy is to pharmacologically enhance the amount of acetylcholine at the NMJ. The second strategy is to modulate the immune system. Most patients require elements of both.

Acetylcholinesterase inhibitors work by inhibiting the actions of acetylcholinesterase—an enzyme present at the NMJ which breaks down acetylcholine. During a normal muscular contraction, acetylcholine released from the presynaptic terminal diffuses across the NMJ combining with acetylcholine receptors on the motor endplate. Conformational change of the receptor causes influx of sodium ions resulting in depolarisation, release of calcium from the sarcoplasmic reticulum, and subsequent muscular contraction. Acetylcholine at the NMJ is broken down by acetylcholinesterase thus terminating its actions and allowing for closure of sodium channels and subsequent repolarisation of the motor end plate. Acetylcholinesterase inhibitors work by inhibiting the breakdown of acetylcholine meaning that more is available to facilitate muscular contraction. They can be categorised according to their duration of action. Edrophonium (see above) is a short-acting drug, useful in diagnosis but too short acting to be useful in treatment. Of the longer-acting agents, pyridostigmine is generally preferred as it has a longer duration of action and less muscarinic side effects than neostigmine [7]. Most patients will respond well to administration of acetylcholinesterase inhibitors.

Second-line treatment involves administration of corticosteroids in an attempt to modulate the immune system and consequently reduce the serum level of antibodies against the acetylcholine receptor. If used, they are generally used in relatively large doses carrying the risk of significant side effects—all of which are well documented in children [15]. Other immunomodulatory drugs such as cyclosporine, azathioprine, and cyclophosphamide are rarely used, due to their significant side-effect profile and limited experience in the paediatric setting.

In the event of an acute deterioration administration of immunoglobulins (IVIGs) or plasma exchange may help to stave off mechanical ventilation [15, 17]. Of the two, plasma exchange is the more effective but also the more laborious to perform, requiring large bore central venous access. For this reason, administration of immunoglobulins is generally the preferred first-line approach, with plasmapheresis available for the more serious cases.

The thymus gland is implicated in a high proportion of children with juvenile MG. Thymectomy has been shown to bring about improvement in symptoms in between 60–90% of patients undergoing the procedure [9, 15]. The effects of thymectomy may take a period of months or even years to bring about such improvements. The procedure is generally performed when children are at least 10 years old [18]. If performed too early the chances of remission are higher and there are theoretical concerns regarding removal of the thymus gland at a period when the immune system is still developing.

## 5. Crises

Myasthenic patients can suffer an acute deterioration in their neuromuscular function sometimes necessitating mechanical ventilation. These episodes are known as crises and take two main forms—myasthenic and cholinergic [11]. A myasthenic crisis is brought about by a relative decrease in the amount of acetylcholine at the NMJ resulting in muscular weakness. It may be precipitated by stress, surgery, or infection, and treatment depends on the administration of anticholinesterases or in more severe cases IVIG or plasma exchange transfusion.

Cholinergic crisis is brought about by excessive administration of acetylcholinesterase inhibitors resulting in an excess of Ach at the motor end plate. In addition to muscular weakness, the child may also demonstrate other cholinergic signs such as sweating, salivation, diarrhea and blurred vision. Treatment is based upon withholding administration of further acetylcholinesterase inhibitors and supportive measures.

The two crises are difficult to distinguish from each other clinically but can be differentiated by the administration of edrophonium. This will bring about an improvement in a myasthenic crisis but worsen a cholinergic one [7]. Evidently, full resuscitative equipment must be on hand prior to its administration.

## 6. Pharmacological Considerations Specific to Anaesthesia

### 6.1. Neuromuscular Blocking Agents

Myasthenic patients have a variable response to muscle relaxants depending on the type of muscle relaxant used. Decreased density of Ach receptors at the motor end plate means that paediatric myasthenic patients may require up to 4 times the calculated dose of suxamethonium in order to bring about a depolarising muscle block [7]. In addition, suxamethonium is metabolised by acetylcholinesterase, and as a result, treatment with acetylcholinesterase inhibitors causes a reduction in its metabolism and a prolongation of its action [19]. For this reason suxamethonium should be avoided in myasthenic patients.

The nondepolarising muscle relaxants on the other hand have a significantly enhanced activity as well as a significantly prolonged duration of action [20]. Unfortunately, the degree to which sensitivity is increased is unpredictable and depends on interaction between disease severity and efficacy of treatment [7, 21–23]. In adults, the most extensively studied muscle relaxants are atracurium and vecuronium. In children, Brown recommended atracurium as the relaxant of choice, due to its metabolism which has been shown to be noncumulative [7]. Rocuronium has been shown to be safe in adults with myasthenia gravis [24], and there are also several reports of its actions being successfully terminated by the administration of sugammadex [25, 26]. Sugammadex negates the need to administer acetylcholinesterase inhibitors to reverse residual neuromuscular blockade and does not carry the associated risks of precipitating a cholinergic crisis. To date, the authors are unaware of any reports in the literature highlighting the use of either rocuronium or sugammadex in paediatric myasthenic patients.

### 6.2. Anaesthetic Agents

Volatile anaesthetic agents are already known to inhibit neuromuscular transmission, and these effects are thought to be exaggerated in myasthenic patients [27–32]. However, no clinically significant postoperative neuromuscular depression has ever been demonstrated with isoflurane, sevoflurane, or desflurane [27, 32, 33]. These neuromuscular deficits are not a feature of propofol, making total intravenous anaesthesia (TIVA); theoretically at least, the technique of choice for these patients [29]. 

Remifentanil is metabolised by nonspecific esterases, and as a result, concern has been expressed regarding a prolonged duration of action in patients treated with acetylcholinesterase inhibitors [34]. This however has not been shown to be the case. Similarly, plasma exchange transfusion is thought to decrease the concentration of plasma esterases. Prolongation in the clearance of remifentanil has however never been demonstrated following plasma exchange transfusion—possibly as it is metabolised to a significant extent by tissue esterases [35].

### 6.3. Anticholinesterases

As already mentioned these drugs bring about a significant prolongation of the duration of action of suxamethonium (and also mivacurium by the same action [36]). In addition, patients receiving preoperative treatment with anticholinesterases may show a decreased response to the administration of neostigmine intraoperatively [12]. This may make reversal of residual neuromuscular blockade even more challenging. Furthermore, intraoperative administration may precipitate cholinergic crisis, potentially confusing the issue of the patient with postoperative weakness.

## 7. Anaesthetic Management

Anaesthetists may become involved in the management of these children for several reasons. Patients may suffer a crisis necessitating institution of mechanical ventilation or the siting of large-bore central venous access to facilitate plasma exchange transfusion. They may require thymectomy, or they may require surgery unrelated to their myasthenia—either in an elective or an emergency situation. Below are some of the general principles that apply to the management of these children, with further detail pertaining to those requiring thymectomy at the end.

### 7.1. Preoperative

Severity of weakness and muscle groups affected should be carefully noted and documented with particular focus on respiratory and bulbar function. Respiratory function tests may be useful in determining the degree of respiratory involvement and the likely requirement for post-operative ventilation. In children, these are frequently not possible, irrespective of whether in the elective or emergency setting. For elective surgery, pre-perative consultation with the child's neurologist should be sought, in order to optimise treatment. Thorough preoperative assessment and pre-optimisation has been shown to improve the frequency of post-operative complications [37]. In the case of severe weakness, pre-operative administration of IVIG or even exchange transfusion may be required to improve neuromuscular function. The child should be placed first on the morning list and have their morning dose of anticholinesterases omitted [38]. The exception to this may be the child with severe weakness who may require continuation of this treatment in order to prevent a significant deterioration.

Clearly in the emergency setting, these are luxuries that one is unlikely to be able to afford. Nevertheless, due consideration should be given towards the level of pre-optimisation that may be achievable within the time constraints imposed by the urgency of surgery.

### 7.2. Anaesthetic Technique

Technique will be influenced significantly by the extent and nature of planned surgery, whether emergency or elective and whether there is risk of a full stomach. For all but the most minor surgery in the stable myasthenic without significant respiratory or bulbar compromise, an endotracheal tube and intermittent positive pressure ventilation are likely to be required. If possible, intubation of the trachea should be performed without the use of muscle relaxants. In the paediatric setting, this is already well described and used frequently. Tracheal intubation with volatiles alone or following an iv induction with propofol and a short-acting opioid are well described in myasthenic children [39–42]. The use of propofol and remifentanil in the form of target-controlled infusion (TCI) is well described in adult myasthenics [43, 44]. TIVA without TCI has also been described in the paediatric literature, but until more recently pump technology has not allowed this technique to be used successfully in children under the age of 16 years [35, 45]. It will be interesting to see whether reports of its use appear in the literature over coming years.

 If muscle relaxants are used, they should be used in smaller doses and should always be used in conjunction with effective neuromuscular monitoring [38]. This should be in the form of a nerve stimulator with an accurate, objective assessment of muscle response such as acceleromyography, mechanomyography, or electromyography. 

Children on high-dose corticosteroids will require supplementation in the perioperative period [13].

The situation of the myasthenic patient requiring rapid sequence induction because of full stomach is a difficult one. Suxamethonium should be avoided because of the reasons already mentioned. Although rocuronium could theoretically be used, determining the dose to give is more difficult. Sugammadex has been used to reverse neuromuscular blockade following administration of 0.5 mg·kg^−1^, but there is no experience of its use following the higher dose required for rapid sequence induction [26]. Intravenous induction with propofol (3 mg·kg^−1^), remifentanil (1.25 mcg·kg^−1^) and lidocaine (1.5 mg·kg^−1^) has been described in a 14-year-old girl requiring rapid sequence induction because of a full stomach [46]. It should be noted that this patient had severe preexisting neuromuscular dysfunction, though the authors noted that excellent intubating conditions were obtained after 60 seconds. There are no other reports in the literature of rapid sequence induction of either adult or paediatric patients.

### 7.3. Postoperative Considerations

Planning for the post-operative period requires meticulous communication between surgeon, anaesthetist, neurologist, and intensivist. Even following a minor procedure in a stable patient, a period of close observation in the recovery area is required to ensure that neuromuscular function—especially pertaining to respiratory and bulbar function—has returned to the preoperative state. Accordingly, the more extensive the surgery, the greater the duration of procedure, and the greater the exposure to drugs that interfere with neuromuscular function, the more important this period becomes. Those at risk of significant reduction in neuromuscular function, or those who have significant respiratory or bulbar impairment preoperatively, may require observation on an intensive care unit. Postoperative ventilation, assuming reasonable respiratory function preoperatively, is rarely required even following extensive surgery [13, 47].

In the event of a significant deterioration in neuromuscular function postoperatively, attention needs to be given towards ascertaining whether one is dealing with a myasthenic or cholinergic crisis. Cholinergic crisis, even if acetylcholinesterase inhibitors have been administered at the end of the procedure, is very rare. If there is any doubt as to the exact nature, edrophonium as opposed to neostigmine should be used due to the rapid onset and offset. Provision should always be made for elective reintubation if respiratory function is deemed inadequate.

### 7.4. Thymectomy

Thymectomy can be performed via an open sternotomy or via a thoracoscopic approach. Thoracoscopic thymectomy is associated with less chest wall trauma and may be associated with lower post-operative morbidity and shorter hospital stay [12, 48]. Surgery itself, however, may be prolonged, and because the mediastinum is accessed via the chest wall, selective one-lung ventilation required. Commonly, the right lung is isolated. A variety of methods have been used, including endobronchial intubation, bronchial blocker, double lumen endotracheal tube, and standard endotracheal tube with carbon dioxide insufflation of the left hemithorax [48]. The potential haemodynamic consequences of such techniques and the effects on gas exchange necessitate invasive arterial monitoring.

Analgesia is another important issue in these patients—especially those undergoing open sternotomy. Epidural analgesia has been described in adult myasthenics undergoing median sternotomy [49]. The requirement for high thoracic block has often deterred paediatric anaesthetists from using this technique due to the possibility of haemodynamic compromise (hypotension and bradycardia) and respiratory embarrassment. More recently, reports have appeared in the literature of using high thoracic epidural to great effect—providing post-operative analgesia without any haemodynamic or respiratory compromise [45]. In cases where epidural analgesia is not used, remifentanil infusion intra-operatively followed by administration of a longer-acting opioid towards the end of the procedure and PCA has been described [38].

Patients should be extubated as soon as possible at the end of the procedure to reduce the incidence of respiratory complications. Prolonged invasive ventilation is associated with greater respiratory morbidity [50]. If the patient is usually taking anticholinesterases but has omitted them on the morning of surgery, a dose of neostigmine is likely to be required towards the end of surgery. Anticholinesterases should be continued in the post-operative period, and dose should be titrated towards effect. Corticosteroids can be weaned down with a view towards stopping [38].

## 8. Conclusions

In this paper we have presented some of the main issues pertaining to the anaesthetist in the management of the child with juvenile MG. We have looked at the underlying pathophysiology of the disease process and how this relates to the applied pharmacology of drugs affecting the neuromuscular junction. We have examined the controversial issue of neuromuscular blocking drugs and explored some of the alternatives to their use. These alternatives often render neuromuscular blocking agents obsolete. In addition, the role of TIVA as the anaesthetic of choice has been explored, and we look forward to hearing about its use in conjunction with TCI more frequently in the future as experience with newer pump technology continues to grow. Finally, we have examined some of the key points in the management of patients presenting for thymectomy. 

Optimal management of any child with myasthenia gravis, irrespective of surgery performed, requires a sound understanding of disease pathophysiology and pharmacology, meticulous planning, and close consultation between surgeon, anaesthetist, and neurologist.
